# Hypoxia Signaling in Parkinson’s Disease: There Is Use in Asking “What HIF?”

**DOI:** 10.3390/biology10080723

**Published:** 2021-07-29

**Authors:** Laura Lestón Pinilla, Aslihan Ugun-Klusek, Sergio Rutella, Luigi A. De Girolamo

**Affiliations:** 1 Interdisciplinary Biomedical Research Centre, Centre for Health, Ageing and Understanding Disease, School of Science & Technology, Nottingham Trent University, Clifton Lane, Nottingham NG11 8NS, UK; aslihan.ugun-klusek@ntu.ac.uk; 2 John van Geest Cancer Research Centre, Centre for Health, Ageing and Understanding Disease, School of Science & Technology, Nottingham Trent University, Clifton Lane, Nottingham NG11 8NS, UK; sergio.rutella@ntu.ac.uk

**Keywords:** Parkinson’s disease, hypoxia, HIF-1, autophagy, proteasome, mitochondria, oxidative stress

## Abstract

**Simple Summary:**

Parkinson’s disease is a neurodegenerative disorder characterized by the death of a specific subset of dopamine-producing neurons. This triggers problems with movement as dopamine is key in regulating motor control. To date, available treatments compensate for dopamine deficiency but are not able to reverse the progressive neuronal cell damage. The exact cause of the loss of these neurons remains to be determined, although it has been linked to environmental factors, genetic predisposition and modifications to vital molecular pathways. Recent evidence shows that events causing reductions in oxygen supply (hypoxia) to these neurons might also be related to PD development. This review explores the link between hypoxia and Parkinson’s disease as well as promising new therapeutic strategies based on HIF-1α, a protein that controls the cellular response to hypoxia. Parkinson’s disease affects around 6 million people, and it constitutes the fastest growing brain disorder worldwide. Therefore, it is of paramount importance to define its causes and investigate new therapies.

**Abstract:**

Hypoxia is a condition characterized by insufficient tissue oxygenation, which results in impaired oxidative energy production. A reduction in cellular oxygen levels induces the stabilization of hypoxia inducible factor α (HIF-1α), master regulator of the molecular response to hypoxia, involved in maintaining cellular homeostasis and driving hypoxic adaptation through the control of gene expression. Due to its high energy requirement, the brain is particularly vulnerable to oxygen shortage. Thus, hypoxic injury can cause significant metabolic changes in neural cell populations, which are associated with neurodegeneration. Recent evidence suggests that regulating HIF-1α may ameliorate the cellular damage in neurodegenerative diseases. Indeed, the hypoxia/HIF-1α signaling pathway has been associated to several processes linked to Parkinson’s disease (PD) including gene mutations, risk factors and molecular pathways such as mitochondrial dysfunction, oxidative stress and protein degradation impairment. This review will explore the impact of hypoxia and HIF-1α signaling on these specific molecular pathways that influence PD development and will evaluate different novel neuroprotective strategies involving HIF-1α stabilization.

## 1. Systemic and Cellular Response to Hypoxia

Oxygen is required for most organisms to produce the energy necessary to fulfil cellular metabolic demands. Thus, when facing hypoxic stress, cells and organisms undergo several molecular and systemic modifications resulting in adaptation and survival.

Low oxygen saturation within the blood circulatory system is sensed by neuron-like glomus cells located in the carotid body [1], whilst neuroepithelial body cells, situated in pulmonary airways, detect oxygen fluctuations in inspired air [2]. Detection of hypoxia triggers a cardiorespiratory response within seconds, consisting of increased pulmonary ventilation and vasoconstriction, elevated heart rate and general vasodilation in order to maximize gas exchange and facilitate oxygen delivery to tissues [3].

**Figure 1:**
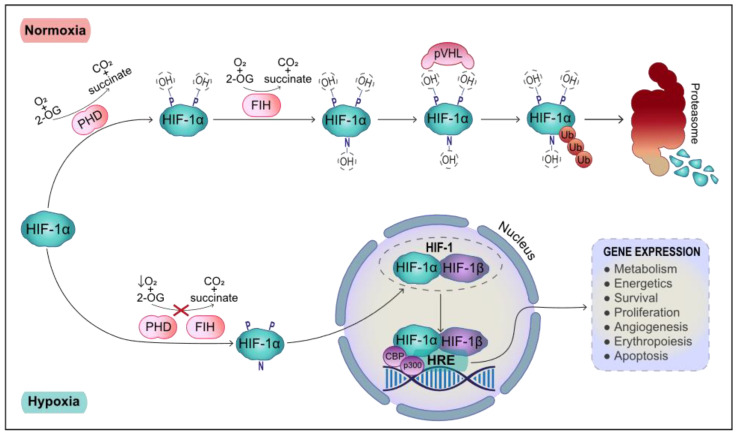
Regulation of HIF-1α stability. In normoxia, HIF-1α is hydroxylated at two proline residues (P402 and P564) by PHDs and at one asparagine residue (N803) by FIHs. Both enzymes utilize oxygen and 2-oxoglutarate (2-OG) and require iron and ascorbate as cofactors. The hydroxyl group linked to the asparagine impairs HIF-1α interaction with its transcriptional cofactors while the hydroxylated prolines are recognized by pVHL, ubiquitin ligase that ubiquitinates HIF-1a, which is consequently degraded by the proteasome. In hypoxia, oxygen shortage hinders PHDs and FIHs activity and therefore HIF-1α is not hydroxylated and can escape degradation. HIF-1 accumulates and is then able to enter the nucleus, forming a complex with HIF-1β referred to as HIF-1. HIF-1 interacts with the transcriptional cofactors CBP and P300 and binds to the HREs present in the promoters of HIF-1 target genes, thereby inducing their expression.

The swift systemic adjustment is followed by a specific cellular response, directed by the principal regulator of the transcriptional response to hypoxia, HIF-1. HIF-1 is a basic helix-loop-helix (bHLH) PAS heterodimer comprising a nuclear constitutively expressed HIF-1β subunit and a HIF-1α subunit, whose expression is precisely modulated by cellular oxygen tension [4] (Figure 1). The bHLH domain mediates HIF-1 dimerization along with the PAS domain and both subunits contain transactivation domains (TAD), necessary to switch on HIF-1 [5]. The α subunit of HIF-1 presents a distinct oxygen-dependent degradation domain (ODDD), which controls its stability [6]. In normoxia, HIF-1α has a remarkably short half-life, as it is continuously synthesized de novo and degraded via the ubiquitin-proteasome system (UPS). HIF-1α is ubiquitinated by Von Hippel Lindau factor (pVHL), the recognition component of an E3 ubiquitin-protein ligase, which interacts with HIF-1α by recognizing two conserved hydroxylated proline residues on its ODDD (P402 and P564 in human HIF-1α) [7]. These prolines are hydroxylated by HIF-1 prolyl hydroxylases (PHDs) that require a significant oxygen concentration to carry out their enzymatic function [8]. Therefore, insufficient oxygen impairs PHD hydroxylation function, disrupting HIF-1α interaction with pVHL and allowing accumulation of HIF-1α, which is then able to enter the nucleus and bind HIF-1β to assemble transcriptionally active HIF-1. In hypoxia, HIF-1 acts as a transcription factor mainly by binding to hypoxia response elements (HRE) in gene promoters, typically containing the consensus sequence 5′-(A/G)CGTG-3′, in the presence of the transcriptional coactivators CBP and p300, facilitating the expression of several genes [9]. However, when oxygen is available, factor inhibiting HIF-1 (FIH), hydroxylates an asparagine residue in the C-TAD of HIF-1α (N803 in human HIF-1α), blocking its interaction with the coactivators, thereby preventing HIF-1 mediated gene expression [10]. Although both FIHs and PHDs, are inactivated by hypoxia, their activity can be inhibited by iron chelators like deferoxamine (DFO) even in the presence of oxygen since both enzymes contain Fe^2+^ at their catalytic sites [11].

As a result of this rigorous HIF-1α regulation, changes to oxygen concentration are precisely translated into adjustments in HIF-1α degradation rate and transcriptional activity.

## 2. Transcriptional Response to Hypoxia: HIF-1 Target Genes

Hypoxia interferes with many crucial molecular pathways, severely compromising cell viability. The role of HIF-1α is to promote the expression of several genes key to cell homeostasis preservation during hypoxic events. To date, over 70 known HIF-1α target genes have been validated and >250 HIF-1α targets have been proposed based on an integrative approach (Table 1) [10,12].

When oxygen levels drop, cellular energy production, which is heavily reliant on oxygen, sharply decreases, provoking an energy crisis. Cells primarily obtain energy from different carbon fuels, such as glucose, that are metabolized and converted to pyruvate and subsequently to acetyl-CoA upon entering the mitochondria. This metabolite enters the tricarboxylic acid (TCA) cycle that is coupled to the electron transport chain (ETC), where the succinate and NADH produced in the TCA cycle will be oxidized. This process provides the high-energy electrons necessary for oxygen breakdown, which generates a proton gradient that powers the ATP synthase. The reduction of oxygen and the synthesis of ATP constitute oxidative phosphorylation (OXPHOS) [13]. In order to maintain cellular ATP levels, HIF-1α elicits a complete metabolic rewiring favoring alternative ATP synthesis via glycolysis instead of through OXPHOS to reduce oxygen consumption. Firstly, HIF-1α will induce the expression of glucose transporters GLUT1 and GLUT3, which facilitate glucose uptake [14]. HIF-1α then stimulates the oxygen-independent energy production through transformation of glucose in lactate via glycolysis by activating gene expression associated to this pathway [15]. Consequently, this reduces oxidative respiration by decreasing pyruvate entry into the TCA cycle [16]. Intriguingly, HIF-1α activity is closely coupled to the TCA cycle, as PHDs and FIHs use the TCA metabolite 2-OG as substrate for their activity, producing succinate as by-product [17]. Thus, high concentrations of 2-OG or its synthetic analogs such as dimethyloxalylglycine (DMOG), can inhibit PHDs and FIHs, promoting normoxic HIF-1α stabilization [18]. Oxygen deficiency also induces a burst in mitochondrial reactive oxygen species (ROS) production, which regulates hypoxic signaling and HIF-1α activation [19]. To mitigate against excess ROS production and further minimize oxygen consumption, the mitochondrial ETC slows down by HIF-1α-mediated downregulation of complexes I and III [20,21]. However, mitochondria that are heavily damaged through hypoxic stress are subsequently targeted for degradation via HIF-1α [22].

HIF-1α promotes cell survival during hypoxia by counteracting the leakage of mitochondrial ROS from the ETC and upregulating oxygen-independent energy production. HIF-1α activates a specific cell-rescue program, inducing the expression of an array of survival factors. HIF-1α facilitates highly efficient transport and delivery of nutrients and oxygen through the expression of erythropoietin (EPO) [23], which stimulates red blood cell production, and vascular endothelial growth factor A (VEGFA) [24], which promotes angiogenesis. In addition, HIF-1α controls the expression of several other angiogenic and survival factors, including angiopoietin-2 and 4 (ANG-2, ANG-4) [25], heme-oxygenase 1 (HO-1) [26], transforming growth factor (TGF-α) [27] and transferrin (TF) [28]. Conversely, if an energy crisis occurs, HIF-1α can impair cell proliferation by regulating the expression of c-myc, p21 and p27 [29], triggering cell cycle arrest, reducing energy consumption and maintaining cell viability. Defective cells, in a similar manner to mitochondria, are eliminated by apoptosis through HIF-1α mediated control of BNIP3, NIX and NOXA [30,31]. In conclusion, HIF-1α-mediated gene expression results in enhanced protection of viable cells and clearance of hypoxia-injured cells.

## 3. Hypoxia in the Nervous System: Links to Neurodegeneration

**Figure 2:**
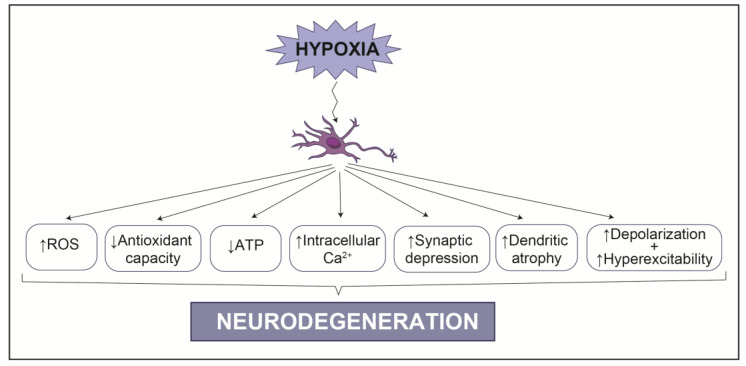
Impact of hypoxia in neuronal homeostasis. Hypoxia can affect several crucial pathways in neurons, facilitating neurodegeneration.

The nervous system requires high energy levels, sustained by an elevated OXPHOS rate, to function properly. Indeed, energy consumption by the mammalian brain accounts for approximately 20% of the oxygen pool present in the body [32]. This oxygen availability is dependent on constant gaseous delivery through the circulation, as the brain oxygen stores are severely limited [33]. Neurons depend upon oxygen to support cellular oxidative respiration as the resultant ATP produced is used to power synaptic action potentials and neurotransmitter trafficking, processes that are crucial for brain function [34]. Thus, it comes as no surprise that the nervous system and particularly the brain are exceptionally sensitive to oxygen shortage. The oxygen supply to the brain can be compromised by deficient oxygen levels in the circulation (hypoxia) or an interruption of blood flow (ischemia), events that can cause death within minutes. Failure of oxygen to reach neurons results in increased free radical generation, reduced antioxidant capacity, rapid ATP depletion and increased intracellular Ca^2+^ levels, followed by generalized synaptic depression, dendritic atrophy and hypoxic depolarization accompanied by hyperexcitability, finally causing death of neural populations (Figure 2) [35]. These changes in the hypoxic brain are analogous with the neurodegenerative and aging brain. Indeed, hypoxic events are linked to the development of neurodegenerative diseases such as Alzheimer’s disease (AD) and Amyotrophic Lateral Sclerosis (ALS) [36,37]. Whilst the mechanism via which hypoxia contributes to ALS progression remains unknown, the impact of hypoxia in AD development has been investigated. AD models have shown that hypoxia can cause amyloid-β accumulation, disruption of Ca^2+^ homeostasis and neuroinflammation [36,37]. However, it has been demonstrated that neurons are able to adapt to hypoxia through activation of the HIF-1α signal transduction pathway, which provides protection against diverse neuronal injuries [38]. Therefore, the broad gene expression profile controlled by HIF-1α has emerged as a valuable neuroprotective candidate due to its ability to regulate cell survival pathways. In fact, targeted induction of moderate HIF-1α levels has neuroprotective benefits in experimental models of neurodegenerative disease [39]. To date, hypoxia has not been directly implicated the pathogenesis of Parkinson’s disease (PD), although therapeutic strategies for PD based on HIF-1α stabilization are beginning to emerge.

## 4. Parkinson’s Disease

PD is the second most prevalent neurodegenerative disease in the world [40], posing a significant burden on healthcare and economic systems. The principal neuropathological hallmark of PD is the loss of dopaminergic (DAergic) neurons in the substantia nigra pars compacta (SNpc) in the midbrain [41]. Dopamine (DA) deficiency arising from this neuronal destruction causes the characteristic PD motor impairments, including bradykinesia, resting tremor, rigidity and postural instability [42]. In most cases, PD origin is idiopathic, resulting from a complex interaction between genetic susceptibility, behavioral influences and environmental factors, although in a small percentage of patients, PD can be caused by specific inherited genetic polymorphisms [43]. The identification of these genes, as well as the discovery of risk factors for PD development, facilitated the identification of key molecular pathways that are disrupted in the neurodegenerative process. Several of these genes and pathways can be linked to—and are regulated by—hypoxia and the HIF-1α signaling cascade.

## 5. Crosstalk between Hypoxia, HIF-1α and PD Related Genes

To date, over 20 PD-linked genetic mutations have been identified, accounting for about 5–10% of PD cases [44]. Although quite rare, these PD-specific mutations have been extensively studied to unravel the signaling pathways responsible for monogenic PD development and assess the frequency of disruption of related molecular cascades in sporadic PD. Intriguingly, a connection exists between several of these genes and the hypoxic response.

α-Synuclein (*PARK1*) is widely expressed in the neuronal cells, specifically in presynaptic terminals, where it controls membrane curvature, thereby contributing to synaptic trafficking, vesicle budding and neurotransmitter release [45]. Impaired α-synuclein function due to mutations or multiplication of the SNCA gene cause autosomal dominant (AD) PD [46]. Indeed, the characteristic Lewy bodies that can appear in the brain of PD patients contain aggregated α-synuclein and are thought to arise from a transition to fibril formation driven by posttranslational modifications and structural rearrangement [45]. This process can be induced by hypoxia, contributing to neuronal dysfunction and death [47]. Interestingly, exogenous α-synuclein oligomers facilitate HIF-1α accumulation in normoxic primary microglia, promoting their migration [48].

Leucine-rich-repeat kinase 2 (*PARK8*; LRRK2) is involved in diverse cellular processes including autophagy, mitochondrial function and cytoskeletal dynamics, consistent with its multiple enzymatic and protein-interacting domains [49]. Mutations in LRRK2 that link to AD PD cluster within two catalytic domains, and often result in increased LRRK2 kinase activity [50]. HIF-1α has emerged as a LRRK2 phosphorylation target in normoxia in human breast cancer cells. This phosphorylated HIF-1α isoform presents greater affinity for its transcriptional cofactor p300, which facilitates the expression of HIF-1α target genes [51]. Conversely, upon traumatic brain injury, HIF-1α can directly bind the LRKK2 promoter and induce LRKK2 expression in the brain, which exacerbates neuronal injury [52].

ATP13A2 (*PARK9*) is a P-type ATPase that localizes to intracellular vesicles and regulates cation homeostasis. This, in turn, impacts on endosomal-lysosomal homeostasis and ensures correct autophagy processing, mitochondrial maintenance and heavy metal detoxification [53]. Loss-of-function mutations that disrupt ATP13A2 activity result in development of autosomal recessive (AR) PD [54]. As ATP13A2 gene promoter contains HREs, HIF-1α stabilization induces ATP13A2 expression in DAergic neurons [55,56]

DJ-1 (*PARK7*) protects cells against oxidative stress through a variety of signaling pathways [57]. Mutations causing defective DJ-1 activity lead to an increased vulnerability to ROS and can trigger early onset AR PD development [58]. Reduced DJ-1 expression correlates with impaired HIF-1α stabilization in hypoxia in several cancer cell lines, MEFs and primary neurons [59,60,61,62]. Similarly, lymphoblasts derived from DJ-1 deficient PD patients also exhibit reduced HIF-1α levels. However, DJ-1 knockout has been related to normoxic HIF-1α stabilization in SH-SY5Y cells and MEFs [63,64].

PTEN-induced kinase 1 (*PARK6*; PINK1) is a serine/threonine kinase mainly involved in maintaining mitochondrial quality and fitness via control of ROS production, oxidative respiration, mitochondrial dynamics and mitobiogenesis. In addition, PINK1 plays a pivotal role in the regulation of mitophagy. During severe mitochondrial stress, mitochondrial depolarization facilitates PINK1 accumulation, which triggers a cascade of events, including Parkin recruitment, leading to mitophagy [65]. Characteristically, PINK1 mutations result in loss-of-function and are linked to juvenile AR PD [66]. Hypoxia can significantly alter mitochondrial homeostasis and OXPHOS. As a consequence, MEFS and primary murine cortical neurons lacking PINK1 present elevated ROS and HIF-1α stabilization [67] but fail to accumulate HIF-1α under hypoxic conditions [68]. Low oxygen environments can alter PINK1 expression, reducing mitobiogenesis in tumor cell lines [69] indicating PINK1 expression can be altered as an adaption to change of oxygen levels.

Parkin (*PARK2*) is a E3 ubiquitin ligase whose interplay with PINK1 controls mitochondrial homeostasis through regulating mitophagy as well as mitochondrial dynamics and biogenesis [70]. Genetic mutations resulting in the loss of Parkin function lead to a failure of mitochondrial quality control and trigger accumulation of defective mitochondria, which can manifest as early onset AR PD [71]. Cell line specific studies show that Parkin can target HIF-1α for degradation in HeLa and breast cancer cell lines, MEFs and human keloids [72,73,74]. In contrast, the Parkin/PINK1 pathway promotes HIF-1α expression in SH-SY5Y and HeLa cells by promoting the degradation of Inhibitory PAS domain protein (IPAS), an effective suppressor of HIF-1α transcription [75]. In glioblastoma-derived cell lines, Parkin knockout facilitates HIF-1α accumulation in normoxia while blocking hypoxic HIF-1α stabilization [76].

Intriguingly, DJ-1, PINK1 and Parkin knockouts show impaired HIF-1α stabilization in hypoxia, indicating that the HIF-1α-mediated response to hypoxic episodes may be defective if loss-of-function mutations in these genes are present. As HIF-1α can directly influence the expression of two PD-related genes, LRRK2 and ATP13A2, through the HREs present in their promoters, and hypoxia can trigger α-synuclein accumulation, it would be of interest to explore the impact of hypoxia and HIF-1α in the expression of other PD-related genes to elucidate possible common mechanisms. Since the aforementioned studies have been performed in a variety of cell lines and animal models, it is of paramount importance to investigate the impact of hypoxic stress and HIF-1α stabilization on PD-related genes in SNpc DAergic neurons and PD animal models in order to adequately establish the role of these pathways in PD.

## 6. Hypoxia and HIF-1α Signaling in Pathways Linked to PD

Genetic mutations in PD can be broadly classified within three distinct pathways: protein clearance, ROS control and mitochondrial function [77]. Dysregulation of these pathways, also found in sporadic PD patients, is believed to be the underlying cause of DAergic neuronal death in the SNpc. Hypoxia significantly affects these specific pathways and HIF-1α-mediated transcription can precisely modulate them. Below we evaluate how hypoxia and HIF-1α signaling can interact with PD-related genes and potentially regulate these key pathways.

### 6.1. Protein Degradation

Protein degradation is carried out by the autophagic pathway and the UPS. Autophagy, divided in macro-autophagy, chaperone-mediated autophagy (CMA) and micro-autophagy, promotes the proteolytic degradation of cellular substrates and organelles through lysosome involvement [78] while the UPS targets proteins by adding ubiquitin residues, which directs damaged and redundant proteins for degradation via the proteasome [79].

Macro-autophagy (referred to as ‘autophagy’) is a homeostatic process comprising the degradation of intracellular components through their engulfment by double membrane vesicles, the autophagosomes, which ultimately fuse with lysosomes, facilitating substrate degradation. The autophagic pathway begins with ULK1 activation to initiate autophagosome formation, driven by LC3-II, Beclin-1, numerous ATG proteins and p62, which acts as an anchor for proteins targeted for degradation [78]. In CMA, selected proteins bind to chaperones forming a complex that is recognized by the lysosomal receptor LAMP2A [78]. Deficiencies in both types of autophagy types have been detected in PD patients’ peripheral blood mononuclear cells [80] and lysosomal vacuole accumulation was detected in DAergic SN neurons [81]. Several of the known PD-related gene mutations can directly and indirectly influence the autophagic process. For example, autophagy impairment facilitates α-synuclein accumulation in the brain [82] and, in turn, α-synuclein aggregation can further hinder autophagy [83]. A loss of function of PINK1 or DJ-1 results in blockade of autophagy and CMA respectively [84,85] while overactivation of LRKK2 reduces both autophagy and CMA [86,87]. Furthermore, inhibition of ATP13A2 function also causes lysosomal dysfunction, accompanied by decreased autophagosome clearance [88,89,90]. Therefore, autophagy induction, as well as modulation of autophagic proteins, have been considered as possible therapeutic strategies for PD, with a primary purpose of promoting α-synuclein clearance [91].

**Figure 3:**
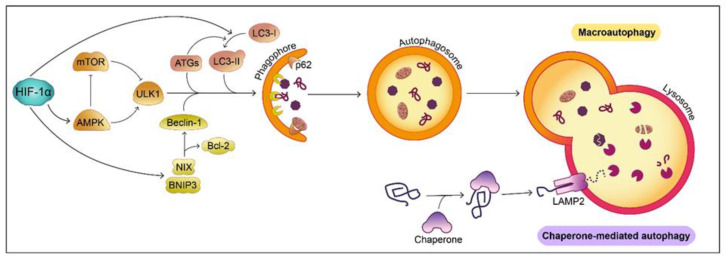
Impact of HIF-1α on autophagy in mammalian cells. Macro-autophagy is controlled by the energy sensor AMPK, induced by HIF-1α. AMPK promotes ULK1 activity while blocking mTOR, inhibitor of ULK1. ULK1 triggers the start of the macroautophagic pathway, facilitating the recruitment of several ATG proteins, Beclin-1 and LC3-II, necessary for the formation of the double membrane, termed phagophore, where the cytoplasmic components targeted for degradation are attached through anchor proteins such as p62. HIF-1α promotes this process by increasing LC3-I lipidation to form LC3-II and inducing the expression of BNIP3 and NIX, which separate Beclin-1 from its inhibitor Bcl-2. Macro-autophagy progresses as the autophagic cargo is enclosed in a double membrane organelle called autophagosome, which fuses with the lysosome, forming the autophagolysosome. The lysosomal enzymes are responsible for degrading the substrates. HIF-1α has not been linked to Chaperone-mediated autophagy, which starts with the unfolding of a damaged proteins by chaperones. The unfolded substrate is detected by the lysosomal LAMP2 receptor, which translocates this substrate to the lysosome for its degradation.

It is well established that hypoxia can trigger autophagy through several mechanisms (Figure 3). HIF-1α upregulates BNIP3/NIX activity, promoting Beclin-1 release from Bcl-2 or Bcl-xL and initiating a signaling cascade [92]. In addition, HIF-1α can facilitate LC3-I conversion to LC3-II leading to the formation of autophagosomes [93]. Both HIF-1α stabilization and hypoxia-mediated ATP depletion activate the master energy sensor of the cell, AMPK, capable of inhibiting mTORC1 and phosphorylating ULK1, triggering autophagy [94,95]. Recent studies point to the involvement of hypoxia-induced ROS in autophagy activation [96]. In addition, hypoxia increases LAMP2A expression, promoting CMA [97]. Basal autophagy is essential for neuronal homeostasis with several studies showing that an autophagy blockade can induce neurodegeneration [98,99]. Despite autophagy playing a key role in maintaining cell viability through substrate clearance, hypoxia-mediated autophagy is considered a double-edged sword, as it is also involved in provoking apoptotic cell death [100]. In the event of a hypoxic episode affecting the brain, hypoxia-induced autophagy can govern cell fate. Upon hypoxic/ischemic (H/I) brain injury, autophagy is activated, promoting either cell survival [97,101,102] or cell death [103,104], dependent on the extent of the hypoxia-mediated damage.

Evidence suggests that the UPS is impaired in PD patients, compromising α-synuclein degradation [105] which accumulates, further hindering proteasome activity [106]. Indeed, treatment with proteasomal inducers promotes α-synuclein clearance in PD models [107,108]. Besides targeting proteins for proteasomal degradation, Parkin can also directly enhance proteasome activity [109]. Contrarily, hypoxic stress reduces proteasome activity [110]. Since HIF-1α is predominantly degraded by the proteasome, inhibition of proteasomal activity leads to its accumulation.

HIF-1α-mediated induction of BNIP3 and the autophagic machinery can compensate for impairments in protein degradation and constitutes a potential therapeutic strategy to resolve the protein aggregation caused by aberrant proteostasis in PD patients.

### 6.2. Mitochondrial Function

Mounting evidence suggests defective mitochondrial function, including biogenesis, dynamics, energy production and mitophagy, in the SN and other selected peripheral tissues of PD patients [111].

**Figure 4:**
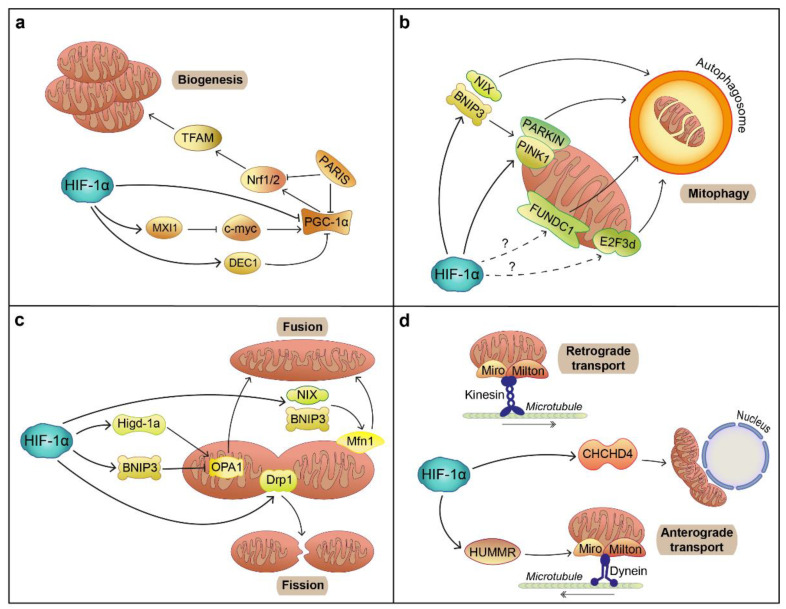
Effect of HIF-1α on mitochondrial number, shape and distribution in mammalian cells. (**a**) Mitochondrial biogenesis. HIF-1α represses mitochondrial biogenesis by preventing activation of the main effector of this process, PGC-1α, either directly or via induction of DEC1, a direct inhibitor of PGC-1α or MXI1, or indirectly through repression of c-myc, which activates PGC-1α. (**b**) Mitophagy. HIF-1α induces mitophagy mainly through BNIP3 and NIX. HIF-1α can also promote mitophagy via PINK1/PARKIN directly or through BNIP3. FUNDC1 and E2F3d mitophagic pathways are induced by hypoxia, although the involvement of HIF-1α has not been demonstrated. (**c**) Mitochondrial dynamics. HIF-1α is capable of inducing fission by promoting Drp1 activity. Fission can be both induced and inhibited by HIF-1α. Fission is promoted by HIF-1α by BNIP3/NIX activation, which promotes Mtf1 function, as well as via HIF-1α-induced Higd-1a, which triggers OPA1 activation. Conversely, BNIP3 can prevent OPA1 activity, hindering mitochondrial fission. (**d**) Mitochondrial transport. HIF-1α induces perinuclear mitochondria accumulation by activating CHCHD4 expression. Additionally, HUMMR, whose levels are controlled by HIF-1α, interacts with Miro to promote anterograde transport of mitochondria.

Mitochondrial biogenesis (mitobiogenesis) is primarily induced upon energy shortage or increased metabolic demand by PGC-1α, a co-transcriptional regulation factor that promotes Nrf1 and Nrf2 transcription, facilitating TFAM expression [112]. Whilst still an area of active research, the PINK1/Parkin pathway appears to participate in this process by regulating the phosphorylation and ubiquitination of PARIS, a known repressor of PGC-1α and Nrf1, in murine SN DAergic neurons and DAergic neurons derived from both hESCs and PD patients’ iPSCs [113,114]. Interestingly, hypoxia induces a stimulatory response to mitigate mitochondrial defects and promotes mitobiogenesis via induction of PGC-1α, Nrf1 or TFAM through pathways involving AMPK, HMGB1 or NOS dependent on cell type [115,116,117]. Hypoxia-induced PGC-1α directly upregulates VEGF and EPO expression [118] and the increased oxygen consumption due to PGC-1α -mediated biogenesis can promote HIF-1α stabilization [119]. Consequently, HIF-1α exerts a compensative response, reducing mitochondrial biogenesis in order to save energy by inhibiting both the expression of PGC-1α either directly or via its repressor DEC1 and c-myc, positive regulator of PGC-1α expression, via upregulation of c-myc inhibitor MXI1 [120,121] (Figure 4a).

Excessive mitochondrial injury triggers mitochondrial membrane depolarization resulting in mitochondrial degradation. Dissipation of membrane potential activates PINK1, which phosphorylates both Parkin and ubiquitin to stimulate their interaction and initiate the Parkin-dependent ubiquitination of mitochondrial substrates. This ultimately triggers mitochondrial clearance through mitophagy [122]. As expected, PD related mutations in Parkin or PINK1 affect mitochondrial degradation and clearance via mitophagy resulting in the accumulation of dysfunctional mitochondria [123]. Despite being able to induce PINK1/Parkin mediated mitophagy in certain conditions [124,125], hypoxia promotes mitophagy via alternative pathways. Low oxygen induces the activation of two mitochondrial membrane proteins, FUNDC1 and E2F3d, capable of interacting with LC3-II and triggering mitophagy [126,127]. Although the involvement of HIF-1α in these pathways has not been studied, HIF-1α can induce mitophagy through the canonical BNIP3/NIX-dependent pathway [22,128], which in turn promotes PINK1/Parkin-mediated mitophagy [129] (Figure 4b).

Mitochondria undergo continuous morphological modifications, including fission, driven by Drp1, and fusion, controlled via mitofusins (Mfn1 and Mfn2) and OPA1 [130]. These processes protect against excessive ROS production and prevent accumulation of defective mitochondria within the cell. LRRK2 directly interacts with Drp1, promoting its recruitment to mitochondria and therefore stimulating fission [131]. Similarly, overexpression of α-synuclein promotes fission independently of Drp-1 [132]. In contrast, the PINK1/Parkin pathway promotes mitochondrial fusion with PINK1/Parkin knockouts exacerbating Drp1-mediated mitochondrial fragmentation [133,134]. Interestingly, upon mitochondrial depolarization, PINK1/Parkin can promote fission prior to mitophagy via regulation of Mfns and Drp1 [135,136]. Hypoxia can promote mitochondrial fission through Drp1 induction via HIF-1α-dependent and independent pathways [137,138]. BNIP3, a HIF-1α target gene, can inhibit OPA1-mediated fusion, causing mitochondrial fragmentation [139]. Conversely, hypoxia facilitates the expression of Hypoxia-induced gene domain protein-1a (Higd-1a), which binds to OPA1 promoting fusion [140] to generate enlarged mitochondria as a compensatory mechanism in a process driven by HIF-1α-mediated expression of BNIP3 and NIX, which promote Mfn1 function [141] (Figure 4c). In addition, hypoxia can induce Parkin-mediated degradation of both Mfns and Drp, potentially suppressing mitochondrial dynamics [142].

Mitochondrial transport is essential in neurons as it responds to regional modifications in ROS and ATP levels by shuttling mitochondria to sites with high energy demand or increased degradation. Therefore, mitochondrial trafficking has been extensively studied in neuronal axons, where retrograde and anterograde transport are regulated by dynein or kinesin respectively [143]. These motor proteins bind mitochondria by means of the adaptor proteins Miro and Milton, which can form a complex with PINK1 [144]. It has been shown that PINK1 can phosphorylate Miro, promoting its degradation via Parkin, thereby inhibiting mitochondrial movement and segregating damaged mitochondria before mitophagy [145]. PD-associated LRRK2 and α-synuclein mutations interfere with mitochondrial transport along the axon [146,147]. Hypoxia has a very specific effect on mitochondrial trafficking, as it induces the accumulation of mitochondria in the perinuclear region. This precisely targeted concentration of mitochondria induces high local ROS production with increased oxygen consumption, which contributes to HIF-1α stabilization and stimulates oxidation of the HRE in the VEGF gene to allow its activation [148]. HIF-1α stabilization through hypoxia facilitates the expression of CHCHD4, further promoting a perinuclear mitochondrial shift [149] while inducing Hypoxia-upregulated mitochondrial movement regulator (HUMMR), which can interact with Miro, fostering anterograde transport [150] (Figure 4d).

**Figure 5:**
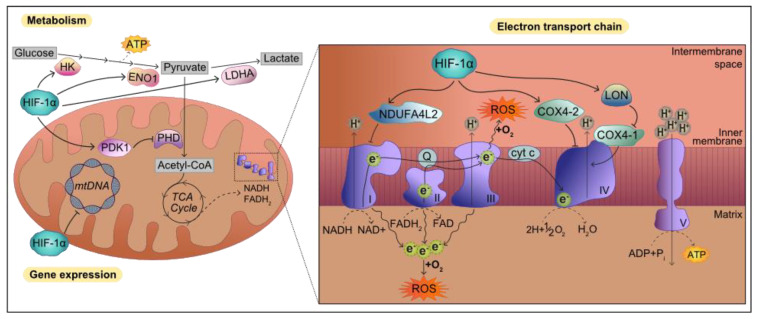
Influence of HIF-1α on mitochondrial energy and ROS production. Due to the limited oxygen availability under hypoxia, HIF-1α promotes alternative ATP synthesis via glycolysis by upregulating the expression of HK and ENO1, which facilitate the conversion of glucose in pyruvate. HIF-1α boosts lactate production from pyruvate by inducing LDH expression while blocking PHD-mediated lactate transformation in Acetyl-CoA through enhancing PDK1 expression. Consequently, the TCA cycle is slowed down and production of ETC substrates NADH and FADH2 is dampened. In order to attenuate ETC activity and reduce ROS, HIF-1α downregulates the activity of complex I by facilitating NDUFA4L2 expression. Additionally, HIF-1α promotes the expression of LON, a protease that degrades cytochrome c oxidase COX4-1 subunit, which is then substituted for COX4-2, whose expression is controlled by HIF-1α. To further diminish ETC activity, HIF-1α blocks mtDNA expression when recruited to mitochondria following oxidative stress damage.

Mitochondrial oxidative respiration and subsequent ATP production have been found to be reduced in PD. PINK1 loss has been linked to impaired activity of several ETC complexes as well as low ATP synthesis, and it has been hypothesized that this respiratory dysfunction causes the collapse of mitochondrial membrane potential [151,152]. As discussed, hypoxia has a heavy impact on mitochondrial energy production, as reduced oxygen availability impairs the ATP synthesis powered by the ETC. Thus, HIF-1α switches off oxidative energy production, favoring glycolytic metabolism. HIF-1α facilitates the expression of hexokinase (HK) and enolase 1 (ENO1), thereby accelerating the production of pyruvate, which is converted to lactate by HIF-1α-induced lactate dehydrogenase (LDHA) [153] instead of being transformed to Acetyl-CoA. This is facilitated by HIF-1α-mediated inhibition of pyruvate dehydrogenase (PDH) activity by promoting the expression of pyruvate dehydrogenase kinase (PDK1) [154] (Figure 5). By lowering Acetyl-CoA levels and decreasing the flux of substrates into the TCA cycle, substrate availability for ETC-mediated ATP synthesis is reduced. Thus, HIF-1α indirectly regulates the deceleration of the ETC. Indeed, HIF-1α induces the expression of NDUFA4L2, which downregulates complex I activity [21] and facilitates the expression of LON, a mitochondrial protease that degrades cytochrome c oxidase COX4-1 subunit. The action of LON facilitates the exchange of COX4-1 for the more efficient COX4-2, whose expression is also controlled by HIF-1α [20] (Figure 5). In addition, HIF-1α can directly translocate to mitochondria upon oxidative damage to downregulate the transcription of mitochondrial genes, further suppressing ETC activity [155] (Figure 5).

### 6.3. Oxidative Stress

ROS are routinely produced by cells, principally through mitochondrial oxidative respiration, and although they have a physiological role in cell signaling, excessive ROS levels can trigger the destruction of intracellular components [156]. To avoid oxidative damage, cells rely on a range of antioxidant enzymes and mechanisms. Accumulation of ROS is associated with increased SNpc DAergic neuron death in PD [157]. Indeed, PD patients exhibit a widespread reduction of antioxidant defenses in the SNpc [158] and lower antioxidant protein levels in peripheral blood [159]. DJ-1 is a major regulator of the antioxidant response, able to induce the expression of several antioxidant genes trough Nrf2 dependent and independent functions [160]. Subsequently, PD-related DJ-1 mutants present reduced antioxidant activity and impaired interaction with the Nrf2/Keap1 pathway [161]. Hypoxic events can exacerbate mitochondrial ROS (mtROS) production, mainly by reducing oxygen availability for complex III [19], but also through modulation of antioxidant gene expression [162,163]. These mtROS seem to contribute to HIF-1α stabilization, although an alternative theory suggests that ETC oxygen consumption has a more significant role in this process. It is possible that HIF-1α stabilization in hypoxia results from a combination of low oxygen and ROS, and the contribution of each could depend on the cell type or other variables. Despite the fact that mtROS-mediated HIF-1α modulation remains a controversial issue, oxidative stress can contribute to HIF-1α accumulation in diverse ways, including the inhibition of PHD activity by Fe^2+^ oxidation [164]. In addition, it has been shown that the application of antioxidants can reduce HIF-1α levels [165] whereas treatment with ROS-producing compounds increases HIF-1 α stabilization even in normoxia [166]. Interestingly, increased ROS due to DJ-1 loss create a pseudohypoxic environment that stabilizes HIF-1α [167]. HIF-1α counteracts ROS production through reorganization of oxygen consumption by the mitochondrial respiratory chain complexes, as well as by downregulating the expression of mtDNA-encoded mRNAs, as previously discussed (Figure 5). Therefore, HIF-1α-mediated control of ROS could prove to be beneficial for neuroprotection against aberrant oxidative stress present in PD.

## 7. PD Risk Factors and Hypoxic Stress

Age is the predominant predisposing risk factor for PD [168]. Whilst aging alone is not responsible for the significant increase in SNpc DAergic neuronal death, it causes reduced DA availability and α-synuclein accumulation that contribute to the dysfunction of several pathways regulating protein degradation, oxidative stress and inflammation [168]. Deregulation of these signaling cascades can be exacerbated by hypoxia via crosstalk with molecular aging mechanisms involving ULK1, AMPK or mTORC1 [169]. Indeed, advancing age is associated with cerebral hypoperfusion which reduces cerebral blood flow (CBF) potentially creating a permanent mild hypoxic state in the brain. Interestingly, the ability of cells to respond to hypoxia declines during aging. Impaired HIF-1α expression and stabilization with a consequent decrease in HIF-1α target genes, accompanied by increased PHD levels was demonstrated by several independent studies in both human and murine aging tissues [170,171,172,173,174]. Of note, HIF-1α has been shown to have an anti-aging role in *C. elegans* [175] and can induce the expression of human telomerase, which protects against cellular senescence [176].

Exposure to pesticides, like rotenone or paraquat, or toxins such as MPTP is an extensively studied risk factor for PD [177]. This has been attributed to their capacity to damage mitochondria by blocking the ETC and producing exacerbated ROS along with their ability to suppress proteasome activity [178]. Indeed, both ROS production and UPS state can modulate HIF-1α state, as discussed previously. Furthermore, complex I inhibitors, including rotenone, are reported to impair HIF-1α stabilization [179]. This could be attributed to the reduction in oxygen consumption within mitochondria due to ETC dysfunction, which elevates cytosolic oxygen levels. Together these mechanisms provide a potential link between hypoxia and PD pathogenesis.

Several other PD risks factors exist but exhibit weaker predictors of PD development. For example, obstructive sleep apnea (OSA) has been identified as predisposing factor for PD development, particularly in the elderly [180]. These patients exhibit increased levels of α-synuclein in their plasma [181]. Interestingly, OSA is characterized by repeated episodes of breathing impairment during sleep, which causes chronic intermittent hypoxia (CIH), a known inducer of oxidative stress in the SN [182].

Traumatic brain injury (TBI), which causes regional brain H/I injury with concomitant neuronal death, is also associated with an increased risk of PD development [183]. The resultant hypoxia can accelerate the accumulation of protein aggregates, contributing to the chronic process of neurodegeneration. This evidence is supported by the identification of elevated α-synuclein levels in cerebrospinal fluid and neuronal axons of TBI patients [184,185].

## 8. Evidence of Hypoxic Injury in the PD Brain

Further indications that hypoxia may play a role in PD pathogenesis come from analysis of hypoxia related events in the PD brain. Chemodetection of systemic hypoxia and resultant initiation of a ventilatory adjustment [186] alongside brain perfusion deficits exist in PD patients, resulting in a reduced oxygen supply to the brain [187]. This could encourage a hypoxic brain environment that can be further depleted of oxygen due to the ventilatory dysfunction arising from reduced DAergic innervation of respiratory muscles in PD patients [188]. Furthermore, SNpc DAergic neurons have long arborized axons, are abundant in synapses and retain spontaneous activity to trigger DA release, features that require continuous energy input, which makes these neurons especially susceptible to factors that compromise energy production, such as hypoxia [189]. Although the selective vulnerability of these neurons to hypoxic stress has not been studied, it appears that H/I events can trigger severe neuronal injury in the SN [190,191]. Intriguingly, HIF-1α is involved in the development and survival of SNpc DAergic neurons via VEGF signaling [192] and can induce the expression of tyrosine hydroxylase (TH), the rate-limiting enzyme for DA synthesis, and the DA transporter (DAT), key proteins for DAergic neuronal function [193].

If hypoxia can influence PD pathogenesis, modulating HIF-1α activity could be essential as a neuroprotective strategy for DAergic survival. However, HIF-1α signaling appears attenuated in PD patients, as gene expression profiling analyses show reduced levels of HIF-1α and its target genes, including VEGF and HK, and upregulation of PHD2 in post-mortem SNpc homogenates of PD patients when compared to age-matched controls [194,195,196]. Several factors could explain this impairment of HIF-1α activation and signaling. For example, the SNpc of PD patients exhibits elevated Fe^2+^ concentrations that contribute to DAergic neuron degeneration [197]. Excess Fe^2+^ promotes PHDs activity, resulting in sustained HIF-1α degradation, which subsequently blocks HIF-1α-mediated expression of iron homeostasis genes such as transferrin, HO-1 and ferroportin [198]. Moreover, the protein levels of HIF-1α transcriptional inhibitor IPAS were increased in the SNpc DAergic neurons of sporadic PD patients [75]. As noted previously, several genetic mutations and disrupted signaling pathways characteristic of PD can block HIF-1α expression and its stabilization. This could have wide ranging effects on HIF-1α mediated signaling in SNpc DAergic neurons and would, not least, functionally impact on parameters of iron homeostasis, antioxidant capacity, mitochondrial fitness, proteostasis and metabolic function.

Thus, insufficient oxygen supply in conjunction with an impaired capacity to trigger the systemic and cellular response to hypoxia may contribute to PD development.

## 9. HIF-1α-Based Therapeutic Strategies for PD

Existing PD medications contain DA precursors, such as levodopa or L-DOPA, DA agonists or monoamine oxidase B (MAO-B) inhibitors, which ameliorate DA deficiency in the SN [199]. Replacement of the lost DA can transiently mitigate PD motor symptoms although progression of the disease continues. Thus, the search for a definitive and effective PD cure is still ongoing. HIF-1α has gained recent attention as potential candidate due to its ability to influence DA production, iron metabolism, mitochondrial function, ROS generation and autophagy. Indeed, a growing number of studies are exploring direct and indirect PHD inhibitors, as well as other molecules that induce HIF-1α stabilization, as novel therapies to modulate aberrant pathways to treat PD (Table 2). Current studies have limitations as some have solely been performed in cell models and mainly target acute cell death and dysfunction. Since PD is a chronic disease, investigations that employ models that mimic the long-term neurodegeneration, such as the rAAV-α-synuclein and the preformed fibril α-synuclein models, should be considered for further research. Use of PD animal models should also be contemplated to test the compounds that have been exclusively investigated in cell culture paradigms.

### 9.1. Indirect PHD Inhibitors

PHDs are indirectly blocked through iron deprivation. The widely used DFO can trigger iron depletion with consequent HIF-1α accumulation and has been shown to be protective against PD features in DAergic cells and PD murine models. In DAergic SH-SY5Y neuroblastoma cells, DFO treatment reduces apoptosis, oxidative stress and ATP loss triggered by 6-OHDA, a compound that selectively destroys DAergic neurons, whilst promoting autophagy through increased autophagolysosome formation and lysosomal enzyme expression [200,201]. DFO treatment also increases the autophagic flow via HIF-1α/Beclin-1, protecting SH-SY5Y cells from rotenone and MPP+, a toxic MPTP metabolite [202]. In the 6-OHDA rat model, intranasal, local or systemic DFO administration has a widespread neuroprotective effect, promoting SN DAergic neuron survival, reducing DA loss in the striatum, controlling ROS production and improving motor behavior [203,204]. Besides attenuating movement deficits, intranasally delivered DFO reduces α-synuclein aggregation in a α-synuclein rAAV rat model [205]. Although the involvement of HIF-1α was not examined in these in vivo studies, DFO-mediated HIF-1α induction drives neuroprotection and motor improvement in a MPTP murine model, and thus HIF-1α is likely to play a role in other in vivo DFO treatment paradigms [206]. Non-invasive intranasal DFO administration represents a promising approach to attenuate the loss of DAergic neurons as DFO readily crosses the blood brain barrier and arrives swiftly to the brain, reducing off-target effects.

Oral administration of M30, an iron chelator, also offers positive results, as it attenuated MPTP-mediated loss of striatal DA and its metabolites, increased TH expression and activity and diminished DAergic neuron death in mice [207]. The neuroprotective role of M30 has been linked to its ability to promote HIF-1α-mediated expression of prosurvival genes [208]. Similarly, the iron chelator clioquinol (CQ) can also stabilize HIF-1α, providing protection against SN DAergic neuron loss caused by MPTP [209]. CQ reduces the formation of α-synuclein inclusions and protects against α-synuclein mediated cell death in the SN in transgenic mice expressing the α-synuclein hA53T mutation [210]. Additionally, CQ mitigates motor deficiencies in a MPTP-induced monkey model of PD. In this model, CQ decreases iron uptake via TfR and efflux through ferroportin while reducing ROS levels [211].

Lactoferrin (Lf), an iron-binding glycoprotein, mitigates MPTP-mediated DAergic neuronal damage and subsequent dyskinesia in mice through reductions in iron uptake and ROS production [212]. Lf elicits its neuroprotective function by upregulating HIF-1α, VEGF, and brain derived growth factor levels and modulating several signaling cascades. Furthermore, experiments performed in SH-SY5Y and MN9D cells showed that stabilization of HIF-1α via Lf was responsible for the induction of TH and several neuroprotective factors leading to increased neuronal viability against MPP+ toxicity. The non-competitive PHD inhibitor FG-0041 can induce HIF-1α stabilization in DAergic cells through iron chelation. FG-0041 induces DA synthesis and metabolism through induction of TH expression in PC12 cells and rat mesencephalic neurons as well as in vivo in the rat striatum [200,213,214]. Furthermore, FG-0041 attenuates 6-OHDA-mediated dissipation of mitochondrial membrane potential and ATP depletion while preserving cell viability in PC12 cells [214]. Together, this data implicates PHD block via iron chelation and subsequently HIF-1α regulation as targets to enhance neuronal function and viability.

### 9.2. Competitive PHD Inhibitors

Competitive PHD inhibition is achieved by directly blocking the interaction of PHDs with its cofactor 2-OG through 2-OG mimicking drugs. One of the first compounds identified was DMOG, which can ameliorate motor impairments and DAergic neuron death associate with parkinsonism caused by manganese toxicity in mice [215]. Increased MPTP-mediated apoptosis of murine cortical neurons, in both WT and DJ-1 KO, can also be rescued by DMOG pretreatment via its ability to stabilize HIF-1α [61]. The anemia drug FG-4592 inhibits PHDs and can protect against MPP+-induced apoptosis in SH-SY5Y cells through the restoration of mitochondrial membrane potential, oxygen consumption, ATP production and mitochondrial biogenesis. Similarly, MPP+-mediated autophagy blockade caused by MPP+ is alleviated by FG-4592 treatment, through a mechanism that enhances LC3-II levels. Furthermore, FG-4592 induces the expression of a range of antioxidant genes including Nrf2, HO-1 and superoxide dismutase, reducing MPP+-mediated ROS production. In MPTP-treated mice, FG-4592 administration restores striatal TH and DA content and preserves DAergic neurons in the SN, alleviating locomotor impairment [216]. In addition, the 2-OG competitive drug JNJ-42041935 can induce HIF-1α stabilization and restore ATP loss provoked by 6-OHDA in SH-SY5Y cells [200]. Similarly, IOX2, which can displace 2-OG from PHDs, suppresses MPTP-induced iron accumulation and apoptosis and restores mitochondrial membrane potential in human iPSC-derived DAergic neurons. These processes depend on HIF-1α activity and ATP13A2 levels [56]. This evidence further highlights how PHD inhibition has the potential to improve neuronal outcomes.

### 9.3. Atypical HIF-1α Inducers

Several compounds have been shown to induce HIF-1α through different mechanisms other than PHD inhibition. In fact, the antiparasitic medication Albendazole (ABZ) promotes HIF-1α and VEGF mRNA expression and diminishes PHD transcription in the presence of rotenone in the rat SN. ABZ counteracts rotenone toxicity by reducing α-synuclein levels, inhibiting the expression of proinflammatory cytokines and enhancing DA synthesis. This ultimately results in reduced rotenone-mediated neuronal injury and motor dysfunction [217]. Protection against rotenone-induced apoptosis is also provided via agmatine, a biogenic amine with neuromodulation properties, which has been shown to promote HIF-1α activation in differentiated SH-SY5Y cells. Additionally, Agmatine restores the loss of mitochondrial membrane potential provoked by rotenone, thereby reducing ROS levels [218]. The hypertension drug hydralazine increases antioxidant capacity and SOD activity and minimizes 6-OHDA induced oxidative stress in SH-SY5Y cells while preserving TH and DAT expression and maintaining cell viability. Interestingly, through an undefined mechanism, Hydralazine can upregulate HIF-1α and VEGF protein levels [219]. Similarly, orexin-A, a neuropeptide released by hypothalamic neurons, promotes HIF-1α accumulation resulting in induction of downstream target genes VEGF and EPO. These are essential for orexin-A mediated mitigation of apoptosis of SH-SY5Y cells triggered by MPP+ [220]. The flavonoid baicalein significantly improves motor performance of MPTP-treated mice. Of interest is that gene expression profiling studies show that baicalein rescues the MPTP-mediated reduction of HIF-1α expression, triggering a neuroprotective effect in these mice [221].

Intriguingly, it appears that PHDs can be inhibited via alternative mechanisms. Upon MPP+ exposure, the hsp90-p23 chaperone complex interacts with PHD2, blocking its degradation. Thus, the p23 inhibitor gedunin reduces PHD2 stabilization, which protects N27 cells and human iPSC-derived DAergic neurons from MPP+-mediated cell death [222]. In addition, adaptaquin (AQ), a selective inhibitor of HIF-PHDs through an unknown mechanism, maintains cell viability by inhibiting the Trip3 prodeath pathway triggered by either MPTP or 6-OHDA in PC12 cells and mice. Interestingly, AQ prevents 6-OHDA-mediated depletion of Parkin levels in PC12 cells. In a mouse model, AQ protected TH+ neurons and their projections to the striatum against 6-OHDA toxicity, thereby maintaining motor performance [223]. Therefore, a growing body of evidence exists that identifies how modulation of PHD and HIF-1α can improve DAergic neuronal function and survival.

## 10. Conclusions

**Figure 6:**
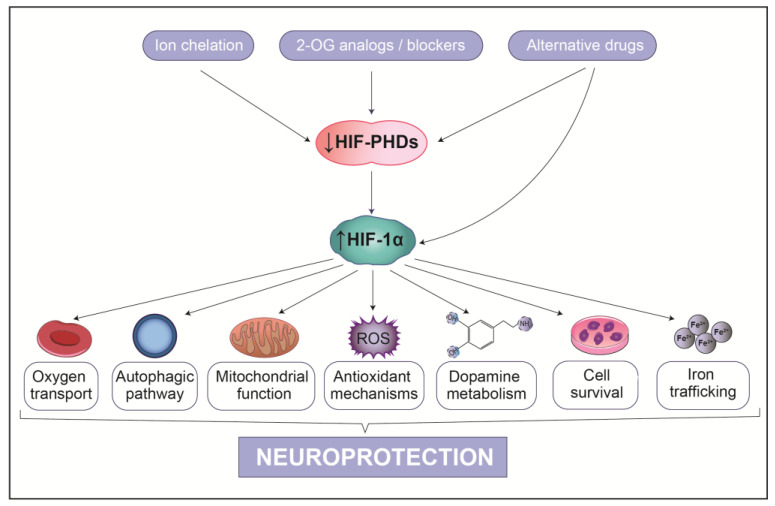
An overview of the potential of HIF-1α stabilization on neuroprotection. Inhibition of HIF-PHDs through several therapeutic strategies leads to HIF-1α stabilization, which facilitates the activation of several neuroprotective pathways.

As the world population ages due to increased life expectancy, the burden of PD is set to rise dramatically. It is therefore imperative to gain a deeper understanding of this disease and determine the exact causes of SNpc DAergic neuronal death. Since hypoxia is already associated with brain disorders, it is plausible to propose that it also impacts on PD. In order to conclusively report the existence of a hypoxic environment in the brain of PD patients, it would be necessary to measure oxygen levels in the PD-damaged SNpc. This is difficult to achieve with current technologies. However, hypoxic stress is at the center of several PD risk factors and is especially detrimental to SNpc DAergic neurons. The involvement of hypoxia in genetic cases of PD is less clear, as the relationship between these genes and hypoxia in SNpc DAergic neurons is still to be elucidated. Nevertheless, these genetic mutations broadly target three main pathways that are profoundly affected by hypoxia and HIF-1α regulation can effectively modulate them. Indeed, pharmacological stimulation of the HIF-1α-mediated response remains a promising therapeutic strategy (Figure 6). HIF-1α activates a wide and diverse transcription program, encompassing genes involved in mitochondrial function, oxidative stress, autophagy, DA production and iron metabolism, which would allow multiple pathways to be targeted that are specifically disrupted in SNpc DAergic neurons in PD. This approach would alleviate neuronal damage through stabilizing HIF-1α levels. However, the broad extent of the HIF-1α transcriptional response is a double-edge sword, as it can cause off-target effects. Concerns exist on the safety of promoting a complete HIF-1α transcriptional response. Consequently, selective induction of downstream effectors would be advantageous. Thus, future research should focus on the design of safe and selective HIF-1α stabilizing drugs and develop appropriate administration strategies to direct these to the brain.

## Figures and Tables

**Table 1:** HIF-1α-target genes. Table summarizing HIF-1α transcriptional targets adapted from Dengler et al., 2014. There are over 70 known direct HIF-1α target genes that function in cell physiological processes. The table is not an exhaustive list of all HIF-1α targets. A more comprehensive list of these genes can be found in Dengler et al., 2014.

HIF-1α-Target Genes						
Oxygen transport and angiogenesis	ADM	ANGPT1	ANGPT2	ANP	BRCP	CP
	CXCL12	EDN1	EPO	FECH	FLK1	FLT1
	GPI PDGFb	HMOX1 PGF	LEP SERPINE1	NOS2 TF	NOS3 TFRC	NOX2 VEGF
Stemness/self-renewal	ADM	EDN1	EPO1	GPI	ID2	IGF2
	PGM	OCT4	TERT	TGFA	VEGF	
Proliferation	CD73	MYC	CTGF	ENG	CDKN1A	CDKN1B
	IGFBP3	ITF	MET	NR4A1	REDD1	RORα4
	STK15	TERT	TGFβ3	WT1		
Apoptosis	BNIP3/3L	NDRG	NIX	NOXA	PP5	MCL1
	NPM					
Oxidative stress	COXAI2	GPX3	HMOX1	LONP1	NDUFA4L	SOD2
Energy metabolism	ALDOA	ALDOC	CA9	COXAI2	ENO1	GAPDH
	GLUT1 LONP1 PFKL TPI	GLUT2 MCT4 PGK1	GPI NDUFA4L PGM	HK1 NHE1 PKM2	HK2 PDK1 TKT	LDHA PFKFB1-4 TKTL2
Mitochondrial homeostasis	BHLHE40	BNIP3/3L	CHCHD4	HIGD1A	MGARP	MXI1
	NIX	PPARGC1				
Autophagy	AMPK	BNIP3/3L	NIX			
Dopamine metabolism	DAT	TH				

**Table 2:** HIF-1α-based therapeutic strategies in PD. Summary of studies involving HIF-1α-stabilizing drugs, including the research models utilized and the mechanisms responsible for HIF-1α-mediated neuroprotection.

Chemical	PD Model	Effects	References
**Indirect PHD inhibitors**			
Deferoxamine (DFO)	Cell model (SH-SY5Y cells)	↑HIF-1α expression ↓Cell death ↓cleaved-PARP, cleaved-CASP3 ↑ATP ↓ROS ↑ Autophagolysosomes ↑Cathepsin, Beclin-1 expression	[200] [201] [202]
	Animal model (6-OHDA rat; α-synuclein rAAV rat)	↑Striatal DA ↑SN DAergic neurons ↓ROS ↑Motor behaviour ↓α-synuclein inclusions	[203] [204] [205]
	Animal model (MPTP mouse)	↑HIF-1α expression ↑SN DAergic neurons ↑DAT expression ↑Bcl-2/Bax ratio ↑GAP43 expression ↑p-ERK/p-p38 MAPK expression ↓p-JNK1/2 expression ↓astrocyte activation ↑Motor behaviour	[206]
M30	Animal model (Mouse, MPTP mouse)	↑HIF-1α expression ↑SN DAergic neurons ↑DA levels ↑TH expression and activity ↑TfR expression ↑Neurotrophic factors ↑Antioxidant enzymes ↑Pro-survival signaling	[207] [208]
Clioquinol (CQ)	Animal model (MPTP mouse; α-synuclein hA53T mouse)	↑HIF-1α expression ↑SN DAergic neurons ↓α-synuclein inclusions	[209] [210]
	Animal model (MPTP monkey)	↑Motor behaviour ↓Non-Motor deficits ↑SN DAergic neurons ↑TH, DAT expression ↓ROS ↓SN iron content ↑AKT /mTOR pathway	[211]
Lactoferrin (Lf)	Cell model (SH-SY5Y cells; MN9D cells)	↑HIF-1α expression ↓Cell death ↓cleaved CASP3 ↑Bcl-2/Bax ratio ↑VEGF, BDNF expression ↑p-ERK expression ↓p-JNK1/2, p-p38 MAPK expression ↑Bcl-2/Bax ratio	[212]
	Animal model (MPTP mouse)	↑HIF-1α expression ↑Motor behaviour ↑SN DAergic neurons ↑TH expression ↓α-synuclein expression ↑Bcl-2/Bax ratio ↓cleaved CASP3 ↓glial activation ↓SN iron content ↑GAP43, BDNF, p-ERK expression ↓p-JNK1/2 and p-p38 MAPK expression	[212]
FG-0041	Cell model (PC12 cells; LUHMES cells; primary rat mesencephalic cells)	↑HIF-1α expression ↓Cell death ↑TH expression and activity ↑DA release ↑ Mitochondrial membrane potential	[200] [213] [214]
	Animal model (rat)	↑TH expression ↑DA levels	[213]
**Competitive PHD inhibitors**			
Dimethyloxallyl Glycine (DMOG)	Cell model (DJ1-KO MPTP primary cortical neurons; SH-SY5Y cells)	↑HIF-1α expression ↓Cell death	[61]
	Animal model (MnCl_2_ mouse)	↑SN DAergic neurons ↑Motor behaviour ↓DNA methylation	[215]
FG-4592	Cell model (SH-SY5Y cells)	↑HIF-1α expression ↓Cell death ↑Bcl-2/Bax ratio ↑TH expression ↑Mitochondrial respiration ↑Mitochondrial membrane potential ↓p62, Cathepsin, LC3-II expression ↑ PGC-1α expression ↓ROS ↑Antioxidant proteins expression	[216]
	Animal model (MPTP mouse)	↑SN DAergic neurons ↑Motor behaviour ↑TH expression ↑DA levels	[216]
JNJ-42041935	Cell model (SH-SY5Y cells; PC12 cells)	↑HIF-1α expression ↑ATP ↑DA release	[200]
IOX2	Cell model (iPSC-derived DAergic neurons; primary mouse mesencephalic cells; differentiated SH-SY5Y cells)	↑HIF-1α expression ↑Mitochondrial membrane potential ↓Iron content ↑ATP13A2 expression	[56]
**Atypical HIF-1α inducers**			
Albendazole (ABZ)	Animal model (rotenone rat)	↑HIF-1α mRNA ↑VEGF mRNA ↑SN DAergic neurons ↑Motor behaviour ↓α-synuclein expression ↑DA levels ↑TH expression ↑TH mRNA, GDNF mRNA ↓NFκB, TNF-α	[217]
Agmantine	Cell model (differentiated SH-SY5Y cells)	↑HIF-1α expression ↑HIF-1α mRNA ↓Cell death ↓CASP3 activity ↓ROS ↑Mitochondrial membrane potential	[218]
Hydralazine	Cell model (SH-SY5Y cells)	↑HIF-1α expression ↑VEGF, TH, DAT expression ↓Cell death ↑Antioxidant capacity	[219]
Orexin-A	Cell model (SH-SY5Y cells)	↑HIF-1α expression ↑EPO, VEGF expression ↓Cell death ↓cleaved-PARP, cleaved-CASP3	[220]
Baicalein	Animal model (MTPT mouse)	↑HIF-1α mRNA ↑Motor behaviour	[221]
Gedunin	Cell model (iPSC-derived DAergic neurons, N27 cells)	↓Cell death	[222]
Adaptaquin (AQ)	Cell model (PC12 cells; primary rat ventral midbrain DAergic neurons)	↓Cell death ↓Trib3, ATF4, CHOP expression/ mRNA ↑Parkin expression	[223]
	Animal model (6-OHDA mouse)	↓Trib3, CHOP mRNA ↑SN DAergic neurons ↑Motor behaviour	

## Data Availability

Not applicable.
